# Prevalence of Suicide Among Adolescents Before and After the COVID-19 Pandemic

**DOI:** 10.7759/cureus.97166

**Published:** 2025-11-18

**Authors:** Viren Parmar, Alejandra Arias Castro, Ishmeet Singh, Gerardo Garcia Santiago, Maheep Singh

**Affiliations:** 1 Medicine, Florida Atlantic University Charles E. Schmidt College of Medicine, Boca Raton, USA; 2 General Medicine, Universidad Autónoma de Guadalajara, Guadalajara, MEX; 3 Department of Science, Stockdale High School, Bakersfield, USA

**Keywords:** covid-19, psychiatry & mental health, stress and depression, suicide risk, world pandemic

## Abstract

This systematic review examines the prevalence of adolescent suicide before and after the COVID-19 pandemic and analyzes associated changes and contributing factors. A literature search was conducted for studies published between 2019 and 2023 in PubMed, Scopus, and Web of Science, focusing on populations aged 12-19 years that reported suicide prevalence both before and during the pandemic. Only peer-reviewed studies meeting the inclusion criteria were analyzed. A total of 20 studies met the criteria and were included. The findings indicate a significant increase in suicidal ideation and suicide attempts among adolescents, particularly females. Major contributing factors included social isolation, academic stress, and reduced access to mental healthcare. Overall, the COVID-19 pandemic has had a substantial negative impact on adolescent mental health. This highlights the urgent need for targeted interventions and strengthened support systems to prevent suicide and promote resilience in this vulnerable population.

## Introduction and background

Introduction

The COVID-19 pandemic, declared a public health emergency of international concern by the World Health Organization in early 2020, marked a turning point in global mental health. Adolescents were disproportionately affected by prolonged social isolation, disrupted educational routines, and heightened family stressors. These circumstances intensified pre-existing mental health problems and triggered new ones. This review synthesizes evidence from 20 key studies to provide a comprehensive understanding of the pandemic’s impact on adolescent suicidal ideation, suicide attempts, and self-harming behaviors.

Effect of COVID-19 on adolescent mental health

The pandemic exacerbated the already fragile mental health of adolescents, with marked increases in anxiety, depression, and psychosomatic complaints. A study conducted in northern Italy revealed persistent anxiety and depression symptoms among adolescents, particularly in females and older age groups. Adolescents with pre-existing psychiatric disorders, such as anxiety and depression, experienced worsening symptoms throughout the pandemic [1].

A systematic review and meta-analysis estimated the global prevalence of suicidal ideation during the pandemic at 12.1%, identifying key contributing factors such as low social support, physical and emotional exhaustion, and poor self-perceived physical health. These factors, especially when combined with academic pressures, were associated with adverse mental-health outcomes among youth [2]. Another meta-analysis reported prevalence rates of 13% for suicidal ideation and 1% for suicide attempts, underscoring the urgent need for targeted mental health interventions. Key risk factors included younger age, female gender, and heightened fear of COVID-19 infection [3]. Taken together, these meta-analyses consistently report comparable prevalence trends, underscoring the robustness of the findings.

Approximately 10% of the global population was reported to have experienced significant psychological distress during lockdowns, with adolescents being particularly vulnerable due to the loss of daily routines and social interaction. Limited social support and financial instability were consistently linked to worsening anxiety and depression [4].

Risk factors and high-risk groups

Multiple studies identified specific risk factors and high-risk populations vulnerable to suicide during the pandemic. Research from China found higher suicidal ideation among males, young adults, frontline workers, and individuals with confirmed or suspected COVID-19 infection or pre-existing psychiatric conditions. Notably, suicidal ideation was more prevalent among middle school students than older adolescents [5]. A retrospective study from King Saud Medical City reported that females and those with prior psychiatric histories were at particularly high risk for suicide attempts during lockdowns. Major stressors included emotional exhaustion, interpersonal conflicts, financial strain, and fear of infection [6]. These findings underscore the importance of early identification and intervention for vulnerable subgroups, including females, children, and individuals with comorbid psychiatric conditions.

Long-term effects of the pandemic continue to persist. A review of adolescents attending an emergency department found sustained increases in suicidal ideation and self-harm behaviors even after restrictions were lifted. The study suggested that the pandemic’s psychological effects may manifest with a delay, particularly among vulnerable populations such as adolescents [7].

Although research has largely emphasized the psychosocial consequences of the pandemic, there is increasing interest in its potential biological and neurological impact. Existing evidence underscores the need for further research into possible neuropsychiatric effects of COVID-19, particularly in developing adolescents [8].

One unintended discovery

Interestingly, a minority of studies reported unexpected mental health improvements among certain adolescents during the pandemic. An editorial in the European Child & Adolescent Psychiatry journal observed that while overall anxiety and depression rates rose, some children experienced better well-being due to stronger family bonds, decreased bullying, longer sleep, and reduced academic pressure [9]. This paradox underscores the complexity of the pandemic’s psychosocial consequences and the need to understand protective as well as risk factors.

Seasonal and social factors

Several studies examined seasonal and social contributors to suicidal behavior during the pandemic. One study comparing suicide attempt rates before and during the pandemic identified the return to school after lockdown as a major risk factor, particularly among females [10]. Social stressors and depressive symptoms appeared to peak during the first semester of the academic year, suggesting that academic reintegration and renewed social exposure may have exacerbated suicidal tendencies.

Implications for mental health interventions

Overall, the evidence highlights the urgent need for broad and targeted mental health interventions to mitigate the long-term impact of the pandemic on adolescents. For example, several studies reported that social isolation, increased family stress, and reduced access to mental health services contributed to rises in self-harm and suicidal thoughts among adolescents. Strengthening school-based mental health programs, community support networks, and public awareness campaigns is essential. Special attention should be given to high-risk populations, particularly females, adolescents with pre-existing mental health conditions, and those exposed to significant life stressors, to prevent future surges in suicidality among youth.

Methods

Literature Search Strategy

A systematic literature search was conducted in PubMed, Scopus, and Web of Science databases using the keywords “adolescent suicide,” “COVID-19,” and “prevalence.” The search included studies published between January 2019 and December 2023. This review adheres to the Preferred Reporting Items for Systematic Reviews and Meta-Analyses (PRISMA) 2020 guidelines for systematic reviews. The completed PRISMA checklist is provided in Supplementary Table S1, and the study selection process is summarized below.

Inclusion and Exclusion Criteria

The inclusion criteria were studies focusing on adolescents aged 12-19 years, articles reporting and discussing suicide prevalence before and during the COVID-19 pandemic, and peer-reviewed journal publications.

The exclusion criteria were studies not published in peer-reviewed journals and articles lacking relevant data on suicide rates or not addressing adolescent high-risk groups.

Data Extraction and Analysis

Data from the included studies were independently extracted by two reviewers and compared across pre- and post-pandemic periods. Any discrepancies were resolved through discussion until consensus was reached. The analysis focused on identifying trends, demographic differences, and contributing factors associated with adolescent suicide. Qualitative synthesis was used due to substantial heterogeneity in study design, methodologies, and reported outcomes across the included studies. Statistical methods reported by the original studies (e.g., Student’s t-test, ANOVA, and Fisher’s exact test) were summarized accordingly.

PRISMA Flow Summary

A total of 265 records were identified through database searches: PubMed (n = 140), Scopus (n = 80), and Web of Science (n = 45). After removing 52 duplicates, 213 records remained for title and abstract screening. Of these, 87 full-text articles were assessed for eligibility, and 20 studies met all inclusion criteria and were included in the qualitative synthesis.

**Figure 1:**
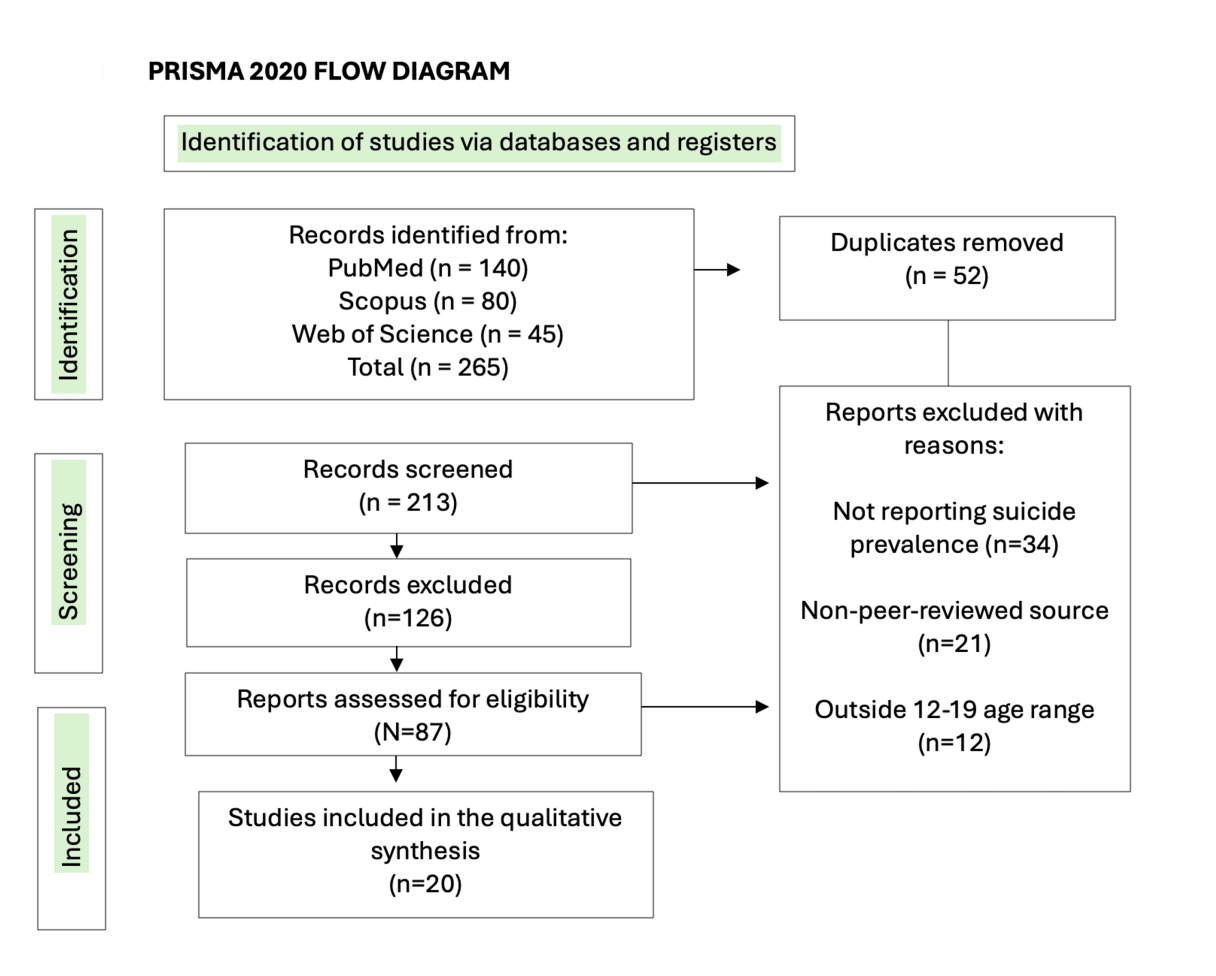
PRISMA (2020) flow diagram summarizing the study selection process. A total of 265 records were identified through database searches: PubMed (n = 140), Scopus (n = 80), and Web of Science (n = 45). After removing 52 duplicates, 213 records remained for title and abstract screening. Of these, 126 were excluded, and 87 full-text articles were assessed for eligibility. Sixty-seven articles were excluded for reasons including lack of suicide prevalence data (n = 34), non-peer-reviewed sources (n = 21), and populations outside the 12–19-year-old range (n = 12). Finally, 20 studies met the inclusion criteria and were included in the qualitative synthesis. PRISMA: Preferred Reporting Items for Systematic Reviews and Meta-Analyses.

The most frequent reasons for exclusion were studies not reporting suicide prevalence (n = 34), non-peer-reviewed sources (n = 21), and populations outside the 12-19-year-old range (n = 12). The complete study selection process is presented in Figure 1.

## Review

Prevalence of suicidal ideation and attempts

During the COVID-19 crisis, suicidal ideation and attempts among adolescents increased worldwide. For clarity, in this review, self-harming behaviors were defined as non-suicidal self-injury (NSSI) without intent to die, whereas suicide attempts were defined as self-injurious actions undertaken with at least some intent to end one’s life. A meta-analysis reported a global prevalence of suicidal ideation of 12.1%, clearly illustrating how the pandemic has profoundly affected mental health across demographics [2]. The marked rise in mental-health crises and suicidal behaviors across different cultural, age, and sex groups warrants further investigation into the risk factors that place specific populations at the greatest risk.

Gender and age differences

According to the literature, both gender and age have been shown to significantly influence suicidal behavior. Girls aged 10-19 years were particularly vulnerable to suicidal thoughts and attempts during the pandemic. Research conducted at King Saud Medical City demonstrated that women attempted suicide more frequently than men during the lockdown period, with stressors primarily involving psychological distress and relationship issues [6].

Further cross-sectional analysis of emergency department records revealed a notably high proportion of adolescent girls, particularly during the first half of the pandemic academic year. The study identified pre-existing depression, social triggers, and younger age as major factors predisposing adolescents to higher suicide attempt rates [10].

Other studies corroborated these findings, showing a gender-specific increase in suicidal behavior among adolescent females. This trend was attributed to heightened vulnerability to social isolation, disrupted social routines, and increased familial stress [11].

A study from China noted that younger adolescents, particularly those aged 15 years or younger, demonstrated a higher prevalence of suicide attempts compared to older groups [5]. Similarly, an article in the International Journal of Mental Health and Addiction suggested that quarantine measures, school closures, limited outdoor activities, disrupted sleep, poor diet, domestic violence, and child abuse collectively contributed to neuropsychiatric disturbances predisposing youth to suicide. Notably, the study highlighted that the loss of school as a social outlet exacerbated psychological distress among children with pre-existing chronic mental or physical health conditions [12].

As presented in Table 1, the distribution of suicide attempts among adolescents differed significantly between pre-pandemic and pandemic semesters, with higher rates observed among females (p < 0.05) [10].

**Table 1:** Number of adolescent suicide attempters by academic semester. Comparison of the number of adolescent suicide attempters between pre-pandemic and pandemic semesters. Statistical analysis was performed using chi-square or Fisher’s exact test. Significance threshold was set at p < 0.05. NS: not significant. Source: Table adapted from Gracia-Liso et al. [10] under the Creative Commons Attribution 4.0 International License.

Variable	First semester, n (%)	Second semester, n (%)	p-value
Pre-pandemic global (girls + boys, n = 47)	31 (66.0)	16 (34.0)	<0.05
Pandemic global (girls + boys, n = 38)	32 (84.2)	6 (15.8)	<0.05
Pre-pandemic girls (n = 40)	26 (65.0)	14 (35.0)	<0.05
Pandemic girls (n = 32)	28 (87.5)	4 (12.5)	<0.05
Pre-pandemic boys (n = 7)	5 (71.4)	2 (28.6)	NS
Pandemic boys (n = 6)	4 (66.7)	2 (33.3)	NS

Risk factors

Several risk factors were consistently identified across the reviewed studies as contributing to the increased prevalence of suicidal ideation and attempts among adolescents. Pre-existing mental health conditions were among the strongest predictors, as youths with prior psychiatric disorders experienced worsening symptoms of depression and anxiety during the pandemic [7]. Social isolation and the lack of social support systems also played a major role, with long-term separation from peers and limited social interactions contributing to deteriorating mental well-being [13]. Academic stress was another major contributor; disruptions in schooling and heightened academic expectations during remote learning increased pressure, frustration, and mental exhaustion among students [10]. Economic and financial stress likewise had a significant impact, as adolescents from low-income households reported higher levels of anxiety and distress related to family instability and job loss [6]. Finally, health concerns, particularly fear of COVID-19 infection in themselves or family members, were frequent sources of distress and uncertainty, especially among those with confirmed or suspected cases [5].

As shown in Table 2, the statistical comparison of sociodemographic characteristics across study years highlights the persistence of several psychosocial stressors during the pandemic period. Statistical analysis was performed using t-tests for continuous variables and chi-square or Fisher’s exact tests for categorical variables, with a significance threshold set at p < 0.05 [1].

**Table 2:** Sociodemographic and pandemic-related characteristics of adolescents (COP-S 2021 vs. COP-S 2022). Significant differences were found for several variables, including family climate, school burden, and social connectedness (p < 0.05). COP-S: Corona and Psyche in South Tyrol Survey; NS: not significant. Source: Table adapted from Barbieri et al. [1] under the Creative Commons Attribution (CC BY) license.

Variable	2021 (n = 1760)	2022 (n = 1885)	p-value
Age (mean ± SD)	14.22 ± 2.34	14.40 ± 2.31	NS
Male, n (%)	857 (48.7)	910 (48.3)	NS
Female, n (%)	903 (51.3)	975 (51.7)	NS
Migration background, n (%)	181 (11.1)	179 (10.3)	NS
Low parental education, n (%)	419 (24.8)	179 (10.3)	<0.05
Single parenthood, n (%)	149 (8.6)	188 (10.0)	NS
Parental mental-health problem, n (%)	53 (3.0)	49 (2.6)	NS
Extended parental workload, n (%)	605 (38.1)	619 (35.6)	NS
General burden (%)	528 (30.2)	22.9 (9.6)	<0.05
Lower family climate, n (%)	464 (26.5)	348 (18.5)	<0.05
Elevated burden at school, n (%)	1151 (65.5)	1087 (57.9)	<0.05
Less contact with friends, n (%)	1067 (60.7)	720 (38.2)	<0.05
Extended use of digital media, n (%)	1214 (69.1)	1049 (55.9)	<0.05

Long-term impact

The long-term effects of the COVID-19 pandemic on adolescent mental health are both enduring and far-reaching. Several studies have noted that the rise in suicidal behaviors and mental health problems continued beyond the initial months of the pandemic. Reports from emergency departments indicated persistent suicidal ideation and self-harm among teenagers even after the acute phase had subsided [7]. Other sources confirmed that the psychological problems observed during the pandemic have remained prevalent, underscoring the need for sustained mental health support and intervention [9]. One article drew parallels between pandemic lockdowns and hikikomori, a Japanese social phenomenon characterized by deliberate social withdrawal. While hikikomori stems from voluntary isolation, lockdowns were mandated by fear of infection and public health restrictions. Nevertheless, both conditions fostered anxiety, sadness, and social avoidance [14]. Prolonged isolation led to an increase in family conflict, excessive internet use, and heightened psychological distress. Although social media and online interactions mitigated some effects of loneliness, they could not replace genuine in-person connections [14].

Protective factors and unexpected findings

Interestingly, some studies documented unexpected improvements in adolescent well-being during the pandemic. For example, 33.2% of children reported enhanced mental health during lockdowns due to reduced bullying, better family relationships, improved sleep quality, and decreased academic stress [9]. However, 33.9% of this same cohort experienced worsened well-being. These contradictory results underscore the complexity of pandemic-related effects and the heterogeneous nature of adolescent resilience. Further research is warranted to identify the protective psychological and environmental factors that enabled some adolescents to maintain or improve their mental health while others declined.

Regional variations

The impact of COVID-19 on adolescent mental health varied significantly across regions and cultural contexts. In Northern Italy, high levels of anxiety and depression were reported, particularly among female adolescents [1]. In China, the prevalence of suicidal ideation was notably elevated among young adults and frontline workers, emphasizing regional differences in stress exposure and coping mechanisms [5]. A cross-sectional study in Portugal and Brazil found that social isolation contributed to higher depression levels in adults and a marked increase in calls to youth helplines reporting anxiety and stress [15]. Quarantined children were more likely to experience acute stress, adjustment disorders, and grief than those who were not isolated. In contrast, data from Queensland, Greece, and Massachusetts revealed stable or even declining depression rates during the pandemic [11], highlighting the importance of cultural, economic, and healthcare-related factors in determining mental health outcomes.

Implications for mental health interventions

Collectively, these findings highlight the need for multifaceted and integrated mental health approaches to address the psychological effects of the COVID-19 pandemic in adolescents. Improving access to mental health care, particularly through telehealth platforms, remains essential [16]. School-based mental health initiatives can help reduce academic stress, foster social reintegration, and support emotional adaptation as schools return to in-person learning [13].

Additionally, the absence of widespread awareness campaigns and insufficient promotion of available mental health resources have contributed to stigma and delayed help-seeking behaviors. Future interventions must focus on gender, age, pre-existing psychiatric conditions, and high-stress life situations as key risk dimensions for developing targeted prevention strategies [13].

Discussion

The COVID-19 pandemic has undoubtedly exacerbated mental health issues among adolescents, leading to a marked increase in suicidal ideation, attempts, and self-harm behaviors. This review synthesizes findings from 20 key studies, providing a comprehensive understanding of how the pandemic has impacted adolescent mental health and highlighting the need for targeted interventions and support systems.

The long-term impact of the COVID-19 pandemic on adolescent mental health is profound and ongoing. Studies have shown that the rise in suicidal thoughts and self-harm behaviors persisted beyond the initial months of the pandemic, necessitating continuous monitoring and support [7]. The sustained mental health challenges faced by adolescents underscore the importance of long-term strategies and interventions to address these issues effectively.

Interestingly, some studies reported unexpected mental health improvements in certain groups of children and adolescents during the pandemic. Improved family relationships, reduced bullying, better sleep patterns, and decreased academic pressure were identified as protective factors that contributed to better well-being for some children [9]. These findings suggest that the pandemic, while posing severe challenges, also created environments that mitigated certain stressors for specific groups, highlighting the complexity of its impact on mental health.

The impact of the COVID-19 pandemic on adolescent mental health varied across different regions and cultural contexts. Studies from Northern Italy, China, and Spain provided diverse perspectives on how regional factors influenced mental health outcomes [1,5,10]. These variations underscore the need for culturally sensitive mental health interventions that consider regional differences in stressors and support systems.

The collective findings from these studies highlight the urgent need for comprehensive and targeted mental health interventions. Enhanced mental health services, including telehealth options, are crucial to provide timely support to those in need. Furthermore, school-based mental health programs can help mitigate academic stress and provide social support, particularly as schools reopen and students readjust to in-person learning [17]. Public health campaigns aimed at raising awareness about mental health issues and available resources are essential to reduce stigma and encourage help-seeking behaviors. Additionally, targeted support for high-risk groups, including females, young adults, individuals with pre-existing mental health conditions, and those facing significant life stressors, is critical to prevent suicidality and promote mental well-being [18].

In light of the post-pandemic mental health challenges, healthcare services need to be adapted with sustainable changes that can help remedy the new challenges faced by adolescents. Some general changes, such as increased remote services to improve access to mental healthcare, increasing community support for school mental health programs, youth helplines tailored to the unique challenges faced by adolescents, and peer support groups to provide emotional assistance, have all been suggested as potential steps in the right direction [19]. Given the increased vulnerability of adolescents and the lasting impact that mental health crises can have on their development, there is a dire need for effective and accessible early mental health resources and interventions. These can aid adolescents by making them aware of their diagnosis and treatment options, as well as providing guidance for their support networks during their development. Aside from healthcare interventions, additional research is warranted regarding the long-term developmental impacts of the pandemic on the youth [20].

Limitations

This review has several limitations that warrant consideration. Heterogeneity in study methodologies, population characteristics, and cultural contexts poses challenges for comparability and may limit the generalizability of the findings. Variation in measurement instruments and follow-up periods further contributes to inconsistency across studies. Additionally, reliance on self-reported data introduces potential recall and reporting biases. Publication bias is also possible, as studies with nonsignificant or negative outcomes may be underrepresented in the published literature. The degree of methodological and outcome heterogeneity prevented the conduct of a quantitative meta-analysis. Finally, the inclusion period (2019-2023) captures only the immediate and early post-pandemic years and may not fully reflect longer-term trends. Even so, adherence to the PRISMA guidelines and a rigorous systematic approach strengthens the credibility of the synthesis.

## Conclusions

The COVID-19 pandemic has exerted a profound and enduring impact on adolescent mental health, reflected in rising rates of suicidal ideation, suicide attempts, and self-harming behaviors. This systematic review, which synthesizes findings from 20 peer-reviewed studies, underscores the multifactorial nature of these outcomes, shaped by demographic, psychosocial, and environmental determinants.

To mitigate these effects, future mental health interventions must be evidence-based, sustainable, and inclusive. Expanding telepsychiatry services, strengthening school-based mental health programs, reinforcing family and peer support networks, and implementing culturally sensitive public awareness campaigns can play a pivotal role in reducing stigma and promoting early intervention. Ensuring accessibility, continuity, and cultural relevance of these programs is essential to safeguard adolescent well-being in the post-pandemic era and to strengthen resilience against future global crises.

## Appendices

**Table 3:** Supplementary Table S1: PRISMA 2020 checklist. Each item indicates where in the manuscript the corresponding information is reported. PRISMA: Preferred Reporting Items for Systematic Reviews and Meta-Analyses.

Section and topic	Checklist item	Location in manuscript
Title	Identify the report as a systematic review.	Title page
Abstract	Provide a structured summary including background, objectives, data sources, eligibility criteria, participants, interventions, and results.	Abstract
Rationale	Describe the rationale for the review in the context of existing knowledge.	Introduction
Objectives	Provide an explicit statement of the objectives being addressed.	Introduction
Eligibility criteria	Specify the inclusion and exclusion criteria for the review.	Methods
Information sources	Describe all information sources used in the search (PubMed, Scopus, Web of Science).	Methods
Search strategy	Present the search strategy used for at least one database, including any filters applied.	Methods
Selection process	Specify the process for selecting studies, including the number of reviewers and screening steps.	Methods, Figure 1 (PRISMA Flow Diagram)
Data collection process	Describe how data were extracted from reports and how discrepancies were resolved.	Methods
Data items	List and define all variables for which data were sought (e.g., author, year, population, and outcomes).	Methods
Study risk of bias assessment	Describe methods used for assessing the risk of bias of included studies.	Methods
Effect measures	Specify the effect measures used for analysis (e.g., frequencies and percentages).	Methods
Synthesis methods	Describe methods used to combine and summarize results.	Methods, Results
Reporting bias assessment	Describe any methods used to assess publication bias.	Methods
Certainty assessment	Describe methods used to assess certainty or confidence in the body of evidence.	Methods
Results of individual studies	Present summary data for each study included in the synthesis.	Results, Table 1 and Table 2
Synthesis of results	Summarize the results of all analyses performed, including comparisons between pre-pandemic and pandemic periods.	Results
Risk of bias in studies	Present risk of bias assessments for each included study, if applicable.	Results
Results of syntheses	Summarize overall findings, consistency, and direction of evidence.	Results
Reporting biases	Present the results of any assessment of reporting bias.	Discussion
Certainty of evidence	Discuss the certainty of evidence for each key outcome.	Discussion
Discussion	Provide a general interpretation of the results in the context of other evidence.	Discussion
Limitations	Discuss limitations of the included evidence and of the review process itself.	Discussion
Conclusions	Provide implications of the results for practice, policy, and future research.	Conclusions
Registration and protocol	Indicate whether a review protocol exists and where it can be accessed.	Methods
Support	Describe sources of financial or non-financial support for the review.	Acknowledgments/Funding Statement
Competing interests	Declare any conflicts of interest of the authors.	Conflict of Interest Statement
